# High Performance MgO-barrier Magnetic Tunnel Junctions for Flexible and Wearable Spintronic Applications

**DOI:** 10.1038/srep42001

**Published:** 2017-02-02

**Authors:** Jun-Yang Chen, Yong-Chang Lau, J. M. D. Coey, Mo Li, Jian-Ping Wang

**Affiliations:** 1 Department of Electrical and Computer Engineering, University of Minnesota, Minneapolis, MN 55455, USA; 2 School of Physics and CRANN, Trinity College, Dublin 2, Ireland

## Abstract

The magnetic tunnel junction (MTJ) using MgO barrier is one of most important building blocks for spintronic devices and has been widely utilized as miniaturized magentic sensors. It could play an important role in wearable medical devices if they can be fabricated on flexible substrates. The required stringent fabrication processes to obtain high quality MgO-barrier MTJs, however, limit its integration with flexible electronics devices. In this work, we have developed a method to fabricate high-performance MgO-barrier MTJs directly onto ultrathin flexible silicon membrane with a thickness of 14 μm and then transfer-and-bond to plastic substrates. Remarkably, such flexible MTJs are fully functional, exhibiting a TMR ratio as high as 190% under bending radii as small as 5 mm. The devices‘ robustness is manifested by its retained excellent performance and unaltered TMR ratio after over 1000 bending cycles. The demonstrated flexible MgO-barrier MTJs opens the door to integrating high-performance spintronic devices in flexible and wearable electronics devices for a plethora of biomedical sensing applications.

Flexible electronics has been a centre of attention in past decades due to the rapid growth of the market, as well as growing scientific interest. Flexibility offers great advantages over conventional rigid electronics, such as light weight, bendable, portable, and potentially foldable devices^1^, which could be integrated onto many kinds of surface. There are prospects for applications such as biological^2^ and wearable devices^3^,^4^. Flexible electronics have benefited from the recent development of organic and inorganic electronics, which are prepared by using thin film technologies or printing. Up to now, remarkable applications based on flexible electronics include displays^5^, organic light-emitting diodes (LED)^6^, organic solar cells^7^, and various kinds of sensors^8^,^9^.

Nowadays, spintronic devices based on giant magnetoresistance (GMR) and magnetic tunnel junctions (MTJs) are widely used for magnetic sensors^10^,^11^, read heads for hard-disk drives^12^, magnetoresistive random access memory (MRAM)^13^ and they have also been proposed for spin logic^14^. The goal of realizing fully-functional flexible spintronic devices is important because it will not only complete the flexible electronics family but also enrich the field of spintronic applications^15^. However, to develop fully-functional spintronic devices on flexible organic substrates is quite challenging. Firstly, most of the organic substrates have a significantly greater surface roughness (at least few nanometers) than silicon, which makes it difficult to grow high-quality magnetic multilayers; secondly, it is difficult to perform microfabrication processes, since heating, physical or chemical etching processes will affect or damage the organic substrates and the magnetic thin films; thirdly, most organic substrates cannot be annealed at high temperature. A few groups have attempted to work on flexible magnetic devices, and flexible magnetic field sensors based on GMR effect have very recently been successfully fabricated and printed onto some kinds of flexible organic substrates^16^,^17^,^18^,^19^,^20^,^21^, Besides, MTJs with Al_2_O_3_ barriers and a big junction size have been grown on flexible organic substrates by using shadow masks^22^,^23^, but they showed rather limited performance. Meanwhile, some processes have been developed to reduce the surface roughness, such as spin-coating a layer of buffer polymer^16^,^17^,^18^,^19^,^20^,^21^,^22^,^23^, which may be good enough for flexible GMR sensors where there is no requirement for a post annealing process, but this is a limiting factor for MTJs with high performance.

MTJs with a crystalline MgO barrier have been intensely studied^24^,^25^ and tunneling magnetoresistance (TMR) as large as 600% at room temperature has been reported in pseudo spin valve stacks^26^. They have become excellent building blocks for different sorts of spintronic devices. So far all of these high performance devices rely on thin-film technologies and are grown on rigid and chemical-mechanically polished silicon substrates with very low surface roughness. Furthermore, to obtain MgO-barrier MTJs exhibiting high TMR, a high temperature vacuum post annealing process is generally required, in order to get good MgO (100) barrier layer and induce crystallization of amporphous CoFeB^26^,^27^. These critical requirements make it almost impossible to fabricate high-quality MgO-barrier MTJs on flexible organic substrates. Most high performance chips nowadays are built on rigid silicon wafers up to half millimeter thick. The wafers are rigid and brittle. It has been found that the silicon becomes flexible and bendable when it is less than about 50 μm thick^28^,^29^,^30^. Meanwhile, transfer-and-bond methods have been successfully used for some kinds of flexible microelectronic devices. They combine the advantages of both conventional devices and flexible materials, and retain good device performance in the flexible form^1^,^2^,^3^,^28^,^29^,^30^,^31^,^32^. Flexible MgO-barrier MTJs should be also possible to make in this way. But to achieve thin enough silicon wafers without any damage to MgO-barrier MTJs is very challenging. Just recently, Loong *et al*. reported the first flexible MgO-barrier MTJs by using a transfer-and-print method^33^. In their methods, a patterned ribbon layer was coated to protect the MTJ device area. And then sacrificial silicon was etched away by an isotropic etching method^34^. An improved performance of flexible MgO-barrier MTJs in a pseudo-spin valve stack was obtained with help of the strain relaxation from SiO_2_ layer.

Here we have developed a different method and successfully demonstrate the intergration of high performance flexible MgO-barrier MTJs directly onto ultrathin flexible silicon membranes. Both MTJ stacks and fabrication methods are different from those in ref. 33. In our process, the backside of the silicon was directly etched in a deep trench etcher system. The final thickness of flexible MgO-barrier MTJs was controlled by the etching time. The flexible MgO-barrier MTJs can be placed onto any kind of nonplanar surface for further testing. The thickness of silicon membrane is about 14 μm, and it can be easily bent to a 3.3 mm radius of curvature without breaking. Further bending to smaller radius of curvature for freestanding silicon membrane become risky since a tiny shear strain will lead to its breakdown. However, it can be further bent to 2 mm radius once it is transferred to a plastic support. The fabricated MgO-barrier MTJs exhibit a room-temperature TMR ratio of up to ~190% with various bending radii, which is much higher than achieved with the previous developed flexible GMR or Al_2_O_3_ barrier MTJs on organic substrates^16^,^17^,^18^,^19^,^20^,^21^,^22^,^23^. These flexible MgO-barrier MTJs open a way to realize high performance spintronic devices in flexible and wearable device applications.

## Results and Discussion

**Figure 1:**
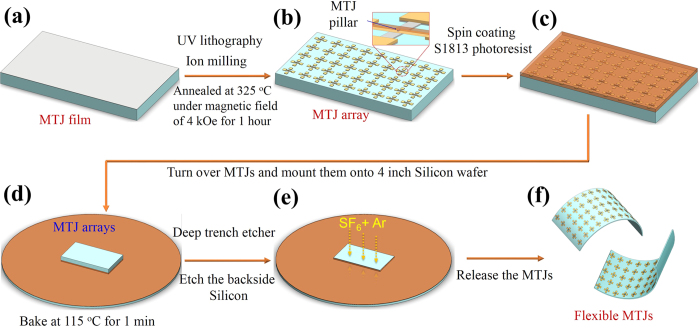
Schematic of the fabrication process for flexible MgO-barrier magnetic tunnel junctions (MTJs). (**a**) MgO-barrier MTJ stacks were grown onto a (150 μm) thin thermally oxidized silicon wafer; (**b**) MgO-barrier MTJs were patterned using standard UV lithography and ion-milling; After post-annealing, (**c**) a layer of S1813 photoresist was spin-coated onto the device surface; (**d**) The sample was turned over, mounted onto a four-inch silicon wafer covered with photoresist; (**e**) A deep trench etching process with SF_6_ and Ar plasma was performed to thin the back side of the silicon; (**f**) After carefully removing the photoresist with acetone, the flexible MgO-barrier MTJs were finally released.

In order to prepare the flexible MgO-barrier MTJs, we used a double-side polished silicon wafer with a norminal thickness of 150 μm having a 300 nm SiO_2_ layer on one side of the substrate. The stacks are prepared at room temperature. Metallic multilayers were grown by DC-magnetron sputtering, and the MgO layer was grown by RF-sputtering from two MgO targets in a target-facing-target gun in a Shamrock sputtering tool^10^,^11^. Figure 1 outlines the fabrication process, and details are provided in the experimental methods section. As shown in Fig. 1a,b, the MTJ stacks were patterned into 4 × 12 μm^2^ rectangular pillars by standard UV lithography and ion milling (Details shown in Figure S2). To obtain a high TMR ratio, high vacuum post-annealing was performed at 325 °C for 1 hour. An external magnetic field of 4 kOe was applied during annealing to set the exchange bias direction. A TMR ratio up to 190% at room temperature is obtained in this step. The TMR ratio here is defined as the magnetic field dependent change of the junction’s resistance: TMR = [*R(H*_ext_) – *R*_sat_]/*R*_sat_ × 100%, where *R*_sat_ is the device’s resistance at saturated state. After that, as illustrated in Fig. 1c–e, a layer of photoresist S1813 was coated onto the MTJ surface to protect the devices, which were then turned over and mounted onto a four inch silicon wafer with photoresist. To thin the silicon wafer from the back, a deep trench etching process was performed, and carefully controlled to obtain the MgO-barrier MTJs samples with a thickness of about 14 μm. Finally, the samples were placed into acetone to remove the photoresist. The flexible MgO-barrier MTJs are finally placed onto a flexible plastic substrate such as Kapton tape to perform the measurements.

**Figure 2:**
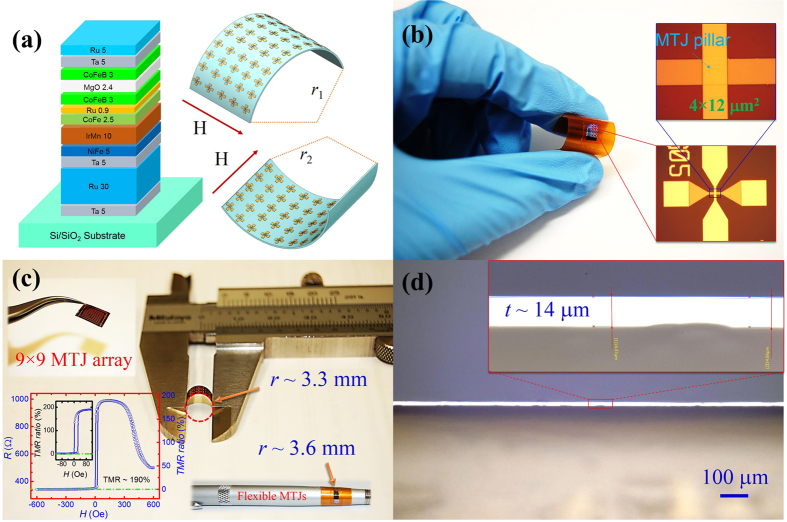
Flexible MgO-barrier MTJ structure, experiment configuration and bending behavior. (**a**) Schematic of the MgO-barrier MTJ stacks used in this work. The external magnetic field was applied along the easy axis and perpendicular to the bending directions. We define the bending radius (*r*_1_) with tensile strain as positive (+) and bending radius (*r*_2_) with compressive strain as negative (−). (**b**) The bending behavior for a real MTJ devices when placed onto a Kapton film. Inset of (**b**) shows the MTJ structures with a pillar size of 4 × 12 μm^2^. (**c**) Free-standing flexible MTJs were bent using vernier calipers, down to a bending radius of 3.3 mm without any damage. Inset of (**c**) shows the TMR full and minor loops with a TMR ratio as high as 190% for an unstressed flexible MTJ device. The resistance-area (*RA*) product is 1.6 × 10^4^ Ωμm^2^. It also shows that this kind of flexible MTJ can be easily be adapted to other curved surface, such as a pen. (**d**) The thickness of the ultrathin silicon substrate for our flexible MTJs is about 14 μm, which is much thinner than the silicon used for convensional silicon chips (500 μm).

The MgO-barrier MTJs have a stack with layer sequences: Ta 5/Ru 30/Ta 5/Ni_81_Fe_19_ (NiFe) 5/Ir_22_Mn_78_ (IrMn) 10/Co_90_Fe_10_ (CoFe) 2.5/Ru 0.9/Co_20_Fe_60_B_20_ (CoFeB) 3/MgO 2.4/CoFeB 3/Ta 5/Ru 5 (thicknesses in nanometers), as shown in Fig. 2a. The Ta/Ru/Ta and Ta/Ru served as the buffer layer and capping layer respectively. A bottom-pinned synthetic antiferromagnetic (SAF) CoFe/Ru/CoFeB stack was used to stabilize the pinned layer. Here the NiFe layer was used to induce a well-textured IrMn (111) layer for improving the exchange bias. The hysteresis loop *M(H*) for unpatterned MTJ stacks was also measured, as shown in Figure S1, which shows the independent switching of CoFeB free layer, NiFe and bottom-pinned SAF layer. The SAF adopts a spin-flop configuration during the magnetization rotation. During the bending measurement, the flexible MgO-barrier MTJs were bent to supply tensile and compressive stress, and the bending radii (*r*_1_, *r*_2_) were positive (+) or negative (−). As shown in the Fig. 2a, an external magnetic field is applied along the easy axis of MTJs and perpendicular to the stress direction during the measurement. This configuration is selected to make sure the external magnetic field always along the easy axis of MTJs during the bending process. A real flexible MgO-barrier MTJ device is shown in Fig. 2b,c, the free-standing MTJ sample is not flat, possibly due to some intrinsic strain, as well as its own weight. It is very fragile. When we carefully compress it with a caliper ruler, it can be bent down to 3.3 mm radius without breaking. Once transferred onto Kapton tape, the flexible MgO-barrier MTJs become stronger and can be easily handled and bent. They can be wrapped around curved surfaces, such as a pen (inset of Fig. 2c, also see additional images shown in Figure S3 and S4). The total thickness of the MTJ sample is about 14 μm, which has been confirmed by optical microscopy, as shown in Fig. 2d. It is possible to further thin down the thickness of samples to several micrometers or even less by carefully controlling the deep trench etching process, and very high flexibility can be obtained in this way. At the end of the whole fabrication process, the TMR ratio does not degrade, but remains about 190%, as illustrated in the inset of Fig. 2c. Both full and minor TMR versus magnetic field loops are shown there.

**Figure 3:**
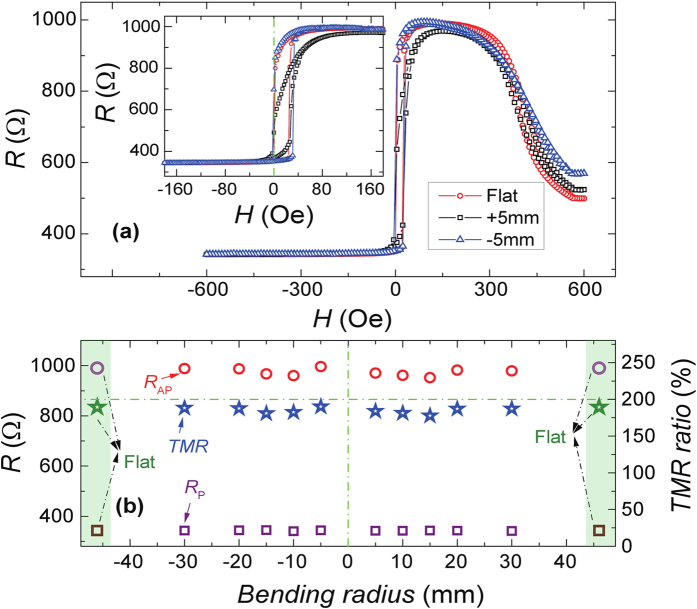
The effect of bending on magneto-transport properties. (**a**) The full and minor TMR loops (inset) with positive (tensile strain) and negative (compressive strain) bending radius of 5 mm. (**b**) Summary of resistance in the parallel (*R*_P_) and antiparallel (*R*_AP_) states, as well as TMR ratios for both positive and negative bending radius from 30 mm to 5 mm. The data for flat configuration were also included as a reference point. The junction size is 4 × 12 μm^2^ and *RA* product is about 1.6 × 10^4^ Ωμm^2^.

The magneto-transport properties as a function of bending radius have been carefully measured at room temperature for the flexible MgO-barrier MTJs samples, as shown in Fig. 3. The sample was pasted on the two sets of curved sample holders with a radius range from 30 mm to 5 mm by using Kapton tape. The sign of the bending radius was defined as positive or negative according to whether the stress on the MTJ was tensile and compressive as shown in the Fig. 2a. The magnetic field was applied along the easy axis and perpendicular to the stress directions. The full and minor TMR versus magnetic field loops with different bending radii are shown in Fig. 3a and its inset for an MTJ pillar with the size of ~ 4 × 12 μm^2^ and resistance area (*RA*) product of ~1.6 × 10^4^ Ωμm^2^. As shown in the inset of Fig. 3a, with positive bending radius of 5 mm, the slope of CoFeB free layer switching decreases; but the slope of CoFeB free layer switching does not decrease and retain shape switching behavior as same as flat configuration with negative bending radius of −5 mm, which is consistent with other reports about stress effect in MTJs^35^,^36^. As shown in the Fig. 3b, with different bending radius, the resistance in the parallel state (*R*_P_) remains almost same, 342.5 ± 1.5 Ω. Only the antiparallel state resistance (*R*_AP_) varies slightly, which results in the changed TMR ratios of 186 ± 6%. The bending radius dependence of *R*_AP_ mainly originates from the stress induced a variation of the anisotropy field of the CoFeB free layer, which slightly changes the antiparallel configuration of MTJs^36^. The similar *R*_P_ suggests that the magnetic-transport through the MgO barrier in our MTJs is robust and almost insenstive to the mechnical strain.

To explain the stress effect in our flexible MgO-barrier MTJs quantitatively, we describe a stress-induced contribution to the anisotropy of the CoFeB free layer: *K*_a,σ_ = 3*λ_s_*σ/2^37^,^38^, and write the stress-induced anisotropy field as: *H*_*a,σ*_ = 3*λ*_s_σ/*μ*_0_*M*_*s*_, so the total anisotropy field *H* will be decrease or increase *H*_a,σ_, with tentile or compressive stress applied. where *σ* is uniaxial stress due to bending; *λ*_s_ is effective magnetostriction coefficient of the CoFeB film; and *M*_s_ is the saturation magnetization of CoFeB. The relation between the stress component (*σ*_*xx*_) and the bending radius (*r*) when the MTJ film thickness is much thinner than the substrate is given by^39^ *σ*_*xx*_ = *Eε* = *Et*/2*r*, so *σ* = *σ*_*yy*_ − *σ*_*xx*_ = −(1 − *ν)σ*_*xx*_ where *t* is the whole thickness of samples (~ substrate thickness); *E* is Young’s modulus, *ε* is the strain and *ν* is Poisson’s ratio. In our flexible MgO-barrier MTJs, the substrate is about 14 μm thick. During our bending test, the minimum bending radius in our measurement is about ±5 mm, so the maximum strain generated is about *ε* ~ ±0.14%. In particular, we will discuss the stress effect based on the TMR minor loops with radius of ±5 mm, as shown in the Fig. 3a. For the silicon substrate, Young’s modulus along [110] is about 169 GPa^40^ and Poisson’s ratio is about 1/3^41^. Assuming that the mechanical strain of the silicon is completely transferred to the MTJ stack, the stress on the CoFeB layer *σ* ~ ±158 MPa for a bending radius of ±5 mm. The magnetostriction coefficient for CoFeB *λ*_s_ ~ 2 × 10^−5 ^42 gives the stress-induced anisotropy *K*_a,σ_ =  ± 4.7 kJ m^−3^. In other words, the effective anisotropy (*K*_eff_) of CoFeB free layer will decrease or increase at most by 4.7 kJ m^−3^ when we bend the MTJ sample to a radius of ±5 mm. By comparing the slope change of CoFeB free layer switching, one can conclude that it is the main origin of the decrease or increase of the slope of CoFeB switching in our TMR loops, as shown in the Fig. 3a. Magnetic-transport properties in MgO-barrier MTJs is highly dependent on the interfacial configuration of CoFeB/MgO, which usually can be determined by measuring bias-voltage dependent d*I*/d*V*^43^,^44^,^45^,^46^. Comparing with bias-dependent first derivative conductance (d*I*/d*V*) in parallel and antiparallel state, as shown in Figure S5, all the curves show similar behaviors with different bending conditions, which may suggest stress applied in our MgO-barrier MTJs is not large enough to change electron band structure. It is also consistent with our TMR behavior with almost no change for different bending conditions (Fig. 3a).

**Figure 4:**
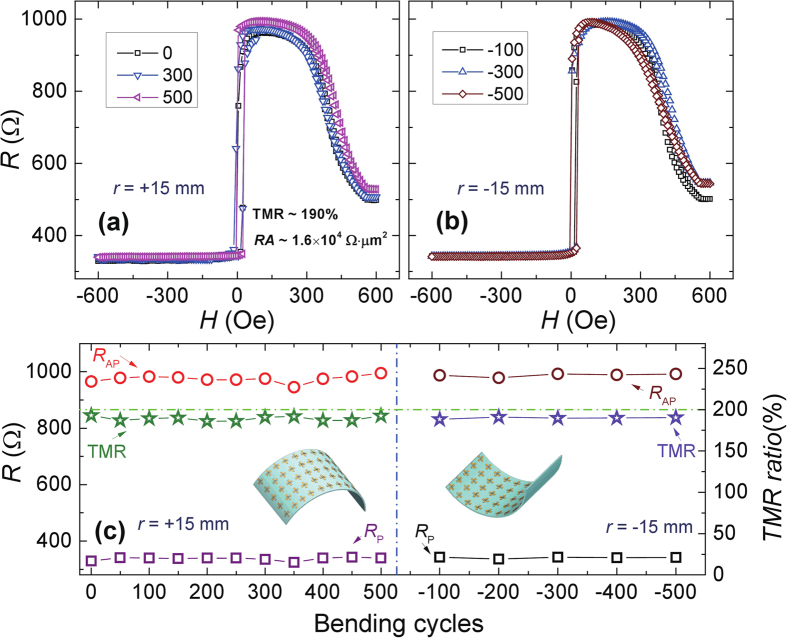
The effect of bending cycles on magneto-transport properties. (**a**) The full TMR loops for different bending cycles with radius of (**a**) 15 mm (tensile strain) and (**b**) −15 mm (compressive strain). The TMR was measured in the flat configuration after certain number of bending cycles for (**a,b**). (**c**) The resistance and TMR ratio of MTJ device after different numbers of bending cycles for a bending radius of 15 mm. After 500 cycles with a radius of 15 mm, the MTJ sample was subject to another 500 cycles with a radius of −15 mm.

Furthermore, the TMR behavior under cyclic bend loading was also investigated. The performance of the devices was not degraded after bending up to 500 times with a radius of 15 mm, or further bending up to another 500 times with a radius of −15 mm. All the TMR loops were measured in the planar configuration after cycling. As shown in Fig. 4a,b, the TMR loops varied slightly for the different bending cycles, which could be attributed to residual stress, as well as a magnetic field direction slightly offset during loading for each measurement. Suprisely, the TMR ratio did not degrade under the 1000 bending cycles, remaining at 189 ± 4%, as shown in Fig. 4c. The resistances in the parallel and antiparallel states also remain same, which indicates high performance and good stability of the flexible MgO-barrier MTJ devices.

## Conclusion

In summary, we have obtained high-performance MgO-barrier MTJs on an ultrathin silicon substrates with thickness down to 14 μm. The whole fabrication procedure did not affect the magnetoresistance. The freestanding flexible MgO-barrier MTJs can be bent down to a radius of 3.3 mm without any damage. Once transferred to Kapton tape, the devices can be further bent down to 2 mm with careful handling. The mechanical stress induced by bending contributes up to ± 4.7 kJ m^−3^ to the effective anisotropy field of the CoFeB layers, which results in a slight variation of *R*_AP_ in the antiparallel state. The TMR ratios remain up to 190% regardless of bending radius. The TMR ratio is unalerted by over 1000 bending cycles. The outstanding performances of the flexible MgO-barrier MTJs make them excellent candidates for high performance on-off flexible magnetic sensors, or even pressure sensors for flexible electronic skins. The work links high-performance spintronics and flexible electronics, which may lead new flexible spintronic device applications.

## Methods

### Preparation of MgO-barrier MTJ stacks

We use a double-side polished silicon wafer with a thickness of 150 μm and 300 nm SiO_2_ on one side as the substrate. The roughness of the substrate is below 0.3 nm. The MgO-barrier MTJ stacks with layer sequences Ta 5/Ru 30/Ta 5/Ni_81_Fe_19_ (NiFe) 5/Ir_22_Mn_78_ (IrMn) 10/Co_90_Fe_10_ 2.5/Ru 0.9/Co_20_Fe_60_B_20_ (CoFeB) 3/MgO 2.4/CoFeB 3/Ta 5/Ru 5 (thicknesses in nanometers) were prepared at room temperature in a modified three-chamber Shamrock sputtering tool. All the metallic layers were prepared by using DC guns, and MgO was grown by RF sputtering using a target-facing-target gun in a different chamber. The base pressure for the metallic films is below 2 × 10^−7^ Torr and for MgO barrier is below 2 × 10^−8^ Torr. A small bias magnetic field of ~ 50 Oe was applied during the metallic film growth, to induce an easy axis in the ferromagnetic layers.

### Fabrication of flexible MgO-barrier MTJ devices

First, the MgO-barrier MTJ stacks were patterned into 4 × 12 μm^2^ junctions using standard UV lithography and ion-milling processes (Shown in Figure S2). The patterned MTJ devices were post-annealed in high vacuum (1 × 10^−6^ Torr) at 325 °C under an external magnetic field of 4 kOe for 1 hour, to set the exchange bias direction, as well as inducing good quality MgO (100) and crystalline CoFe for a high TMR ratio. Secondly, after testing the TMR behavior, a layer of photoresist S1813 was spin-coated onto the surface at a speed of 2000 rpm to protect the devices. Meanwhile, a four inch silicon wafer was also spin-coating with a layer of S1813 at the same speed, and the MTJ devices were turned over and mounted onto this silicon wafer surface and baked together at 115 °C for 1 min on a hot plate. Thirdly, the wafers with MTJs were placed into a deep trench etcher system. The back side of the silicon was carefully etched away with SF_6_ and Ar plasma to reach our required thickness. Finally, the wafers with MTJs were put into acetone solution for a while to remove the residual S1813 photoresist. The flexible MgO-barrier MTJs were released and carefully taken out to rinse with de-ionized water and dried with N_2_ gas.

### Magnetic and transport properties measurement

The magnetic properties of post-annealed unpatterned MgO-barrier MTJ stacks were measured at room temperature using a vibrating sample magnetometer (VSM). For the transport measurement, the flexible MgO-barrier MTJs were mounted onto Kapton tape first to enhance their strength and placed onto two kinds of curved sample holders with different radii ranging from 30 mm to 5 mm. The magneto-transport properties of MTJ samples were measured with a constant dc current of 10 μA during bending at room temperature. The external magnetic field was applied along the easy axis of MTJ devices and perpendicular to the mechanical stress directions.

## Additional Information

**How to cite this article:** Chen, J.-Y. *et al*. High Performance MgO-barrier Magnetic Tunnel Junctions for Flexible and Wearable Spintronic Applications. *Sci. Rep.* **7**, 42001; doi: 10.1038/srep42001 (2017).

**Publisher's note:** Springer Nature remains neutral with regard to jurisdictional claims in published maps and institutional affiliations.

## Supplementary Material

Supplementary Information
