# Supplementary material for: High Performance MgO-barrier Magnetic Tunnel Junctions for Flexible and Wearable Spintronic Applications
Source: Sci Rep. 2017 Feb 2;7:42001. doi: 10.1038/srep42001 (PMC5288802; doi:10.1038/srep42001)
Supplement: Supplementary Information [file srep42001-s1.pdf]

Supplementary Information

High Performance MgO-barrier Magnetic Tunnel Junctions for Flexible and Wearable Spintronic Applications

Jun-Yang Chen[1,*], Yong-Chang Lau[2,†], J. M. D. Coey[2], Mo Li[1,*] and Jian-Ping Wang[1,*]

[1]*Department of Electrical and Computer Engineering, University of Minnesota, Minneapolis, MN 55455, USA*

[2]*School of Physics and CRANN, Trinity College, Dublin 2, Ireland*

[*]**Corresponding authors**. Email: (jychen@umn.edu; moli@umn.edu; jpwang@umn.edu)

[†]**Present address**: National Institute for Materials Science, Tsukuba 305-0047, Japan

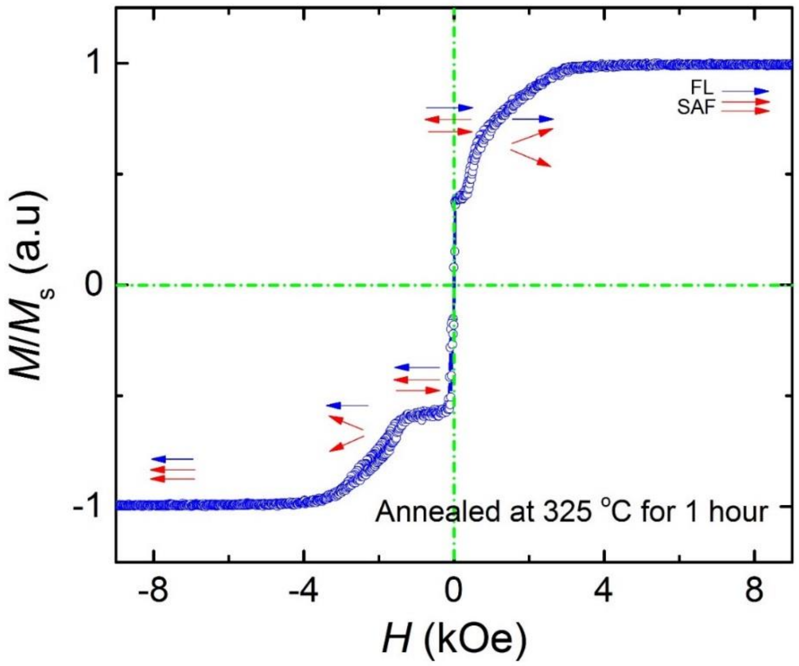

Figure S1. M-H loop of MTJ film which was measured along the easy axis of bottom pinned layers at room temperature. The MTJ films have been annealed at 325 °C under a magnetic field of 4 kOe for 1 hour, which has same annealing condition as the MTJ devices. The arrows in the figures are used to illustrate the magnetic moment directions for free layer (FL) and synthetic antiferromagnetic (SAF) layers under sweeping in-plane external magnetic field.

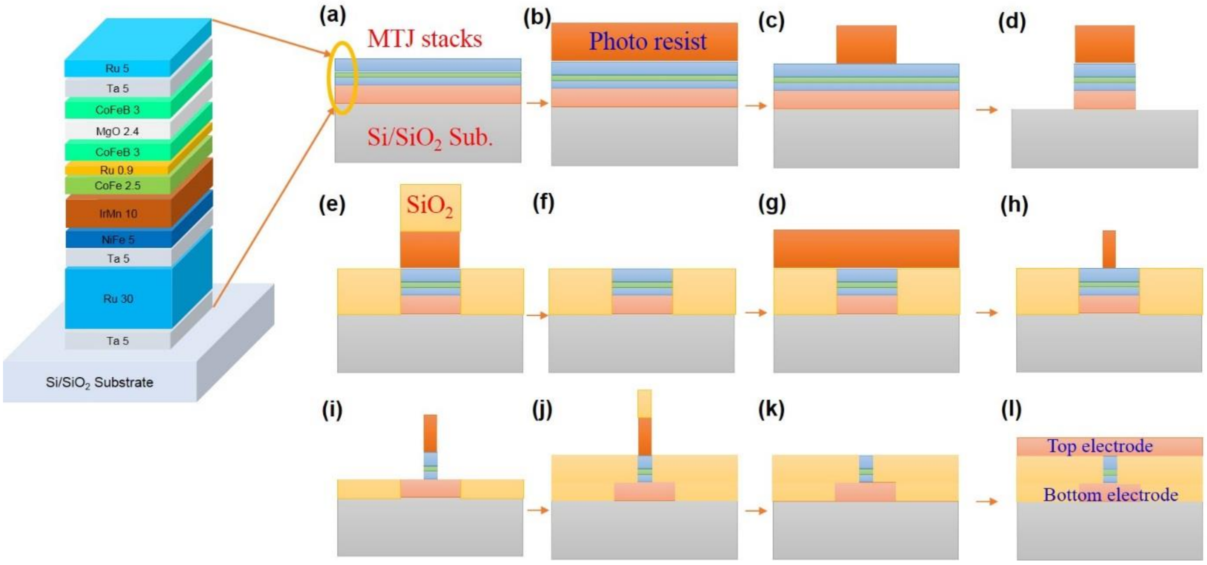

Figure S2. MgO-barrier MTJ fabrication process used in our experiment for all of our MTJs, which is same as the traditional MgO-barrier MTJ fabrication process. These process have been ignored in the Figure 1a-b. Sample dimensions are not drawn to scale.

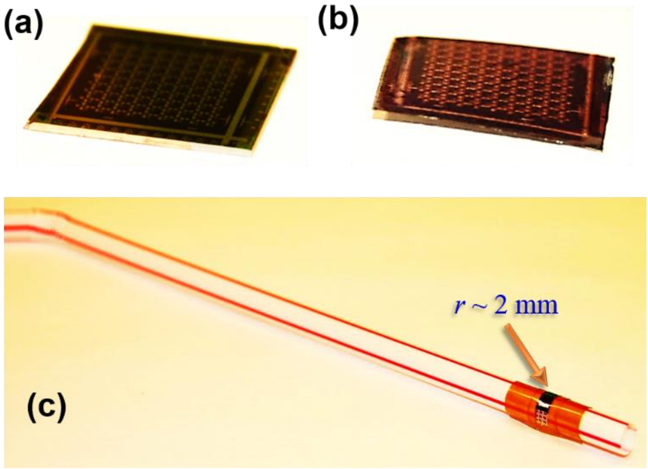

Figure S3. (a) A standard MgO-barrier MTJ devices fabricated onto convesional thermal-oxide silicon wafer (500 μm). (b) A flexible MgO-barrier MTJ devices developed in this work onto ultrathin silicon membrane (~ 14 μm). It has already become unflat due to some intrinsic strain. (c) Our flexible MgO-barrier MTJ devices can even be placed onto a straw, which has a radius about 2 mm. It has potential applications in the field of bio-detections.

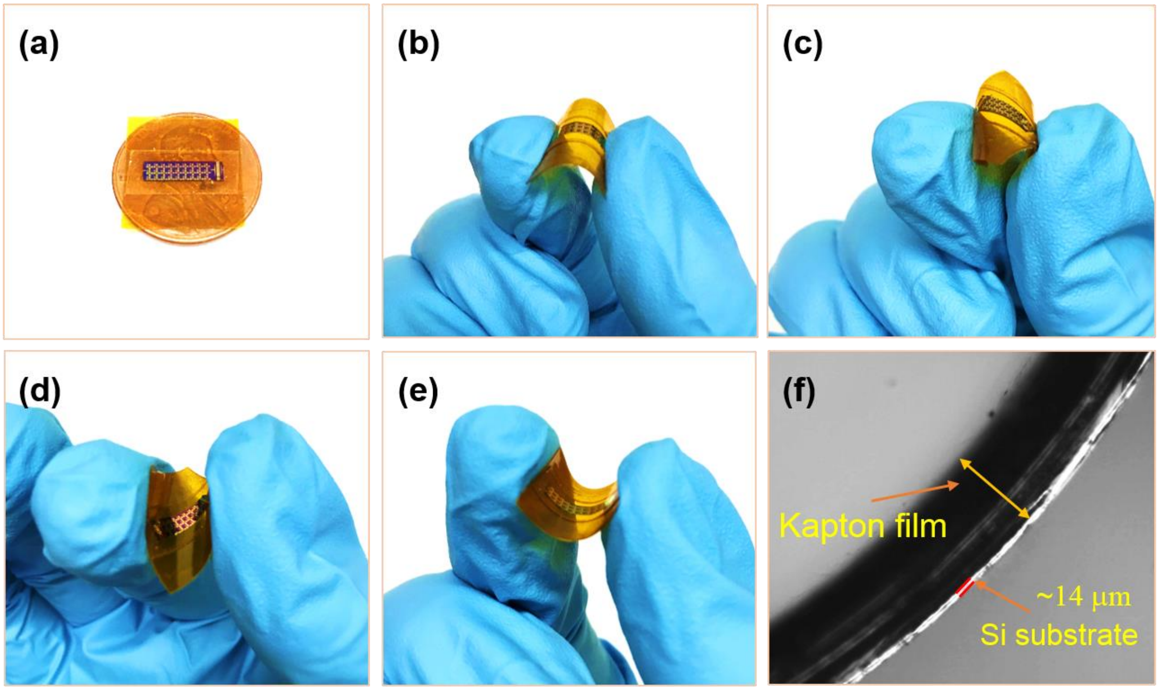

Figure S4. (a) One flexible MgO-barrier MTJs sample used in our experiment. (b)-(e) The MgO-barrier MTJ samples under different kind of bending conditions. (f) The side-view of silicon substrate with MTJs mounted onto Kapton film under bending.

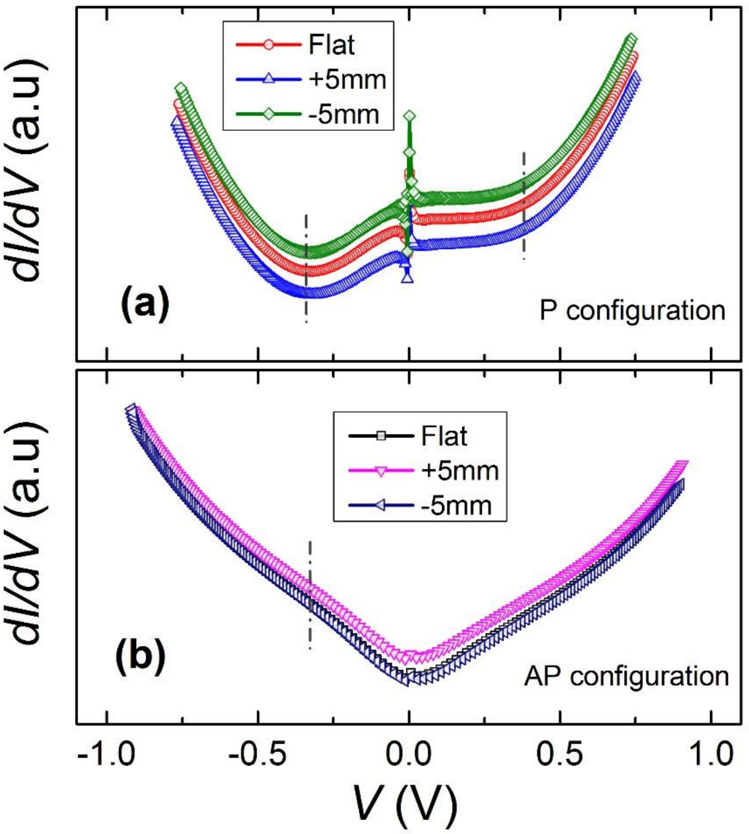

Figure S5. The bias voltage dependent first derivative conductance (d*I*/d*V*) in (a) parallel (P) and (b) antiparallel (AP) magnetization configuration for one of flexible MgO-barrier MTJ devices with different bending conditions. In our measurement, the positive current represents current flow from bottom electrode to top electrode.
